# Three-dimensional reconstructions of intrahepatic bile duct tubulogenesis in human liver

**DOI:** 10.1186/1471-213X-11-56

**Published:** 2011-09-26

**Authors:** Peter S Vestentoft, Peter Jelnes, Branden M Hopkinson, Ben Vainer, Kjeld Møllgård, Bjørn Quistorff, Hanne C Bisgaard

**Affiliations:** 1 Department of Cellular and Molecular Medicine, Faculty of Health Sciences, University of Copenhagen, The Panum Institute, Blegdamsvej 3B, DK-2200 Copenhagen N, Denmark; 2 Department of Biomedical Sciences, Faculty of Health Sciences, University of Copenhagen, The Panum Institute, Blegdamsvej 3B, DK-2200 Copenhagen N, Denmark; 3 Department of Pathology, Rigshospitalet, Copenhagen University Hospital, Blegdamsvej 9, DK-2100 Copenhagen Ø, Denmark

## Abstract

**Background:**

During liver development, intrahepatic bile ducts are thought to arise by a unique asymmetric mode of cholangiocyte tubulogenesis characterized by a series of remodeling stages. Moreover, in liver diseases, cells lining the Canals of Hering can proliferate and generate new hepatic tissue. The aim of this study was to develop protocols for three-dimensional visualization of protein expression, hepatic portal structures and human hepatic cholangiocyte tubulogenesis.

**Results:**

Protocols were developed to digitally visualize portal vessel branching and protein expression of hepatic cell lineage and extracellular matrix deposition markers in three dimensions. Samples from human prenatal livers ranging from 7 weeks + 2 days to 15½ weeks post conception as well as adult normal and acetaminophen intoxicated liver were used. The markers included cytokeratins (CK) 7 and 19, the epithelial cell adhesion molecule (EpCAM), hepatocyte paraffin 1 (HepPar1), sex determining region Y (SRY)-box 9 (SOX9), laminin, nestin, and aquaporin 1 (AQP1).

Digital three-dimensional reconstructions using CK19 as a single marker protein disclosed a fine network of CK19 positive cells in the biliary tree in normal liver and in the extensive ductular reactions originating from intrahepatic bile ducts and branching into the parenchyma of the acetaminophen intoxicated liver. In the developing human liver, three-dimensional reconstructions using multiple marker proteins confirmed that the human intrahepatic biliary tree forms through several developmental stages involving an initial transition of primitive hepatocytes into cholangiocytes shaping the ductal plate followed by a process of maturation and remodeling where the intrahepatic biliary tree develops through an asymmetrical form of cholangiocyte tubulogenesis.

**Conclusions:**

The developed protocols provide a novel and sophisticated three-dimensional visualization of vessels and protein expression in human liver during development and disease.

## Background

In the human embryo, the earliest morphological manifestation of the developing liver is the hepatic diverticulum. This structure is discernible as a thickening of endodermal cells in the embryonic foregut at the 17 somite stage, corresponding to 3 weeks + 5 days post conception [1]. Under the influence of mesodermal signaling, cords of endodermal cells expand from the cephalic part of the hepatic diverticulum into the septum transversum [2,3]. The parenchymal cords anastomose around pre-existing endothelial-lined spaces, increase in mass and become more organized at the expense of the septum transversum that eventually forms the liver capsule [2].

Several theories exist regarding the development of intrahepatic bile ducts from this early stage liver. Current understanding of the process is that primitive hepatocytes in contact with the mesenchyme surrounding developing hepatic portal veins form a single-layered structure known as the ductal plate. The ductal plate becomes bi-layered with a parenchymal and a mesenchymal facing sheet, respectively. The ductal plate consists of cuboidal cells with increased immunoreactivity for epithelial intermediate filaments such as cytokeratins (CK) 8, CK18 and CK19, relative to the surrounding parenchymal cells [4,5]. Slit-like lumina form between the two layers of cells that migrate into the portal mesenchyme to form mature intrahepatic bile ducts [6,7]. Thus, development of intrahepatic bile ducts by cholangiocyte tubulogenesis can be suggested to occur through a series of remodeling stages, i.e. the "ductal plate" stage, the "remodeling bile duct" stage, and the "remodeled bile duct" stage [8-10].

In adult liver, the intrahepatic bile duct is connected with the hepatocytic canaliculi via the Canal of Hering. Current consensus is that the Canal of Hering being the most distal part of the biliary tree constitutes the hepatic progenitor cell niche, a protective microenvironment in which hepatic progenitors reside [11-14]. In prenatal liver, the ductal plates are suggested to not only constitute the hepatic progenitor niche, but also to be directly antecedent to the canal of Hering [11,13]. The postnatal liver has considerable inherent regenerative capacity. Following acute injury, the tissue mass is restored by mitotic division of mature hepatocytes and cholangiocytes. However, when this capacity is compromised during massive or chronic injury hepatic progenitor cells are recruited to restore a functional liver [15-18].

When analyzing human liver sections histologically, a somewhat constant architecture of hepatocytes, vessels and bile ducts can be demonstrated. This monotonous histological appearance actually reflects a highly complex tissue architecture that is not well understood and frequently debated [19-21]. Therefore, gaining knowledge of the mechanisms involved in human cholangiocyte differentiation is expected to enhance fundamental understanding of cholangiocyte tubulogenesis. This is obtained by visualizing not only ductal plate formation and the remarkable remodeling of the biliary tree during liver development, but also the branching network in adult normal and diseased liver.

The aims of the present study were to develop protocols for digital reconstruction of the human intrahepatic biliary system and the portal vessels in three dimensions (3D) from 2 dimensional photographs of serially sectioned liver, and to describe human intrahepatic cholangiocyte tubulogenesis. These 3D-protocols were based on two approaches: volumetric rendering and surface rendering through image segmentation. Volumetric renderings are constructed by calculating the brightness and color from each volumetric pixel element (voxel) based on the voxel color value and the voxel transparency [22]. Thereby, a three dimensional picture can be created displaying the actual sections in colors without the need for segmenting the objects of interest on each photograph. Surface rendering based on image segmentation involves the process of outlining the objects of interest, thereby assigning each pixel to a certain object or structure and creating vectors for visualizing the objects in three dimensions.

To prove the validity of this 3D-approach in studies of human liver biology, we stained serial sections from adult normal and acetaminophen intoxicated livers with the cholangiocyte marker CK19 recapitulating a previous non-digitized 3D-study by Theise et al. [14]. Next, in order to describe the structural development of the intrahepatic biliary tree, a unique collection of liver from adults as well as human embryos and fetuses ranging from 5 weeks + 6 days to 15½ weeks post conception were stained with markers previously used to identify the cholangiocytic or hepatocytic lineages in the adult liver [4,23-26]. These markers included CK7 and CK19, epithelial cell adhesion molecule (EpCAM), hepatocyte paraffin 1 (HepPar1), sex-determining region Y (SRY)-box 9 (SOX9) and aquaporin 1 (AQP1). In addition, laminin and nestin were used as markers of extracellular matrix deposition during tubulogenesis. By means of the developed 3D-protocols, digital reconstructions were created of the three developmental stages, i.e. the "ductal plate", the "remodeling bile duct" and the "remodeled bile duct" stages, with emphasis on the localization of CK7, CK19, EpCAM, SOX9 and AQP1.

The digitized 3D-reconstruction study successfully depicted the spatial expression of marker proteins and the distribution of vessels in portal areas of the developing and adult human liver. It also demonstrated the tubular formation of cholangiocytes into a branching network that will eventually form the human intrahepatic biliary tree. The findings support the theory that human intrahepatic biliary tree development proceeds by an asymmetrical tubulogenesis, which has recently been described in mice [26]. Finally, it was shown that the ductular reaction characteristic of acetaminophen intoxication forms a tortuous network of interconnected CK19 positive biliary structures extending from the bile duct epithelium into the parenchyma, as originally described by Theise et al. [14].

## Methods

### Human tissue specimens

Samples of human embryonic, fetal and adult liver tissues (5 weeks + 6 days to 15½ weeks post conception, n = 42) were obtained either from the archives of Department of Cellular and Molecular Medicine (ICMM) at the University of Copenhagen or from legal abortions performed at Department of Obstetrics & Gynaecology, Frederiksberg Hospital, Copenhagen. Informed consent was obtained from all contributing women according to and approved by The Regional Committee on Biomedical Research Ethics of Copenhagen and Frederiksberg (KF (01) 258206). Crown-rump lengths were measured on the embryos and fetuses and their ages were estimated in days or weeks following conception (a complete list of liver samples and their characteristics can be found in Table 1). The livers were fixed in 4% buffered formalin for at least 18 hrs and subsequently embedded in paraffin.

**Table 1:** Investigated human specimens

Embryos		Fetuses and adults	
**Crown Rump Length (mm)**	**Weeks + days post conception**	**Crown Rump Length (mm)**	**Weeks post conception**

13	5 + 6	32	8
15	6 + 1	34	8½
15	6 + 1	35	8½
16	6 + 2	36	8½
18	6 + 5	38	8½
18	6 + 5	41	9
20	7 + 0	42	9
21	7 + 1	44	9½
21	7 + 1	44	9½
23	7 + 2	48	10
23	7 + 2	48	10
27	7 + 5	60	10½
27	7 + 5	73	11½
27	7 + 5	92	12½
28	7 + 6	95	12½
29	8 + 0	102	13½
		103	13½
		107	13½
		110	14
		120	15
		125	15½
		55 years	Adult*
		58 years	Adult*
		n/a	Adult*
		n/a	Adult*
		28 years	Acetaminophen

A complete list of the human tissue specimens obtained from embryos, fetuses and adults. Crown Rump Lengths measured on the aborted specimens were used for estimation of their gestational ages. *Adult liver tissue originated from one unused donor liver or from non-involved areas of livers resected for metastatic colorectal disease or with primary hepatocellular carcinomas.

### Immunohistochemistry

Five-μm thick liver sections were used and antigen retrieval was applied when necessary. Experimental details on the utilized primary antibodies and the conditions for antigen retrieval are provided in Table 2. Binding of primary antibodies was visualized using "Envision+ System-HRP Labelled Polymers" technique (Dako, Glostrup, Denmark) and diaminobenzidine (DAB) (Sigma, MI, USA). Sections were counterstained with Mayer's hematoxylin.

**Table 2:** Applied antibodies and retrieval systems

Primary antibodies	Cat. # (clone)	Manufacturer	Dilution	Retrieval system
CK7	M7018 (OV-TL 12/30)	Dako	1:50	Retrieval solution, High pH*
CK19	M0888 (RCK108)	Dako	1:50	Retrieval solution, High pH*
HepPar1	M7158 (OCH1E5)	Dako	1:35	Retrieval solution, High pH*
EpCAM	M0804 (BER-EP4)	Dako	1:200	Retrieval solution*
Laminin	Z0097	Dako	1:500	Proteinase K, ready to use^+^
SOX9	HPA001758	Sigma	1:25	Retrieval solution*
AQP1	AB3065	Chemicon	1:1200	
Nestin	MAB5326 (10C2)	Chemicon	1:300	

*Sections were boiled in Dako retrieval solution (S3307/S1699) for 10 minutes in a microwave oven and let to cool to room temperature for 20 minutes

^+^Sections incubated with Dako proteinase K (S3020) for 2½ minutes at room temperature.

Utilized secondary antibodies from Dako were anti-mouse (K4000) or anti-rabbit (K4002).

### Computer-assisted three dimensional-reconstructions

**Figure 1:**
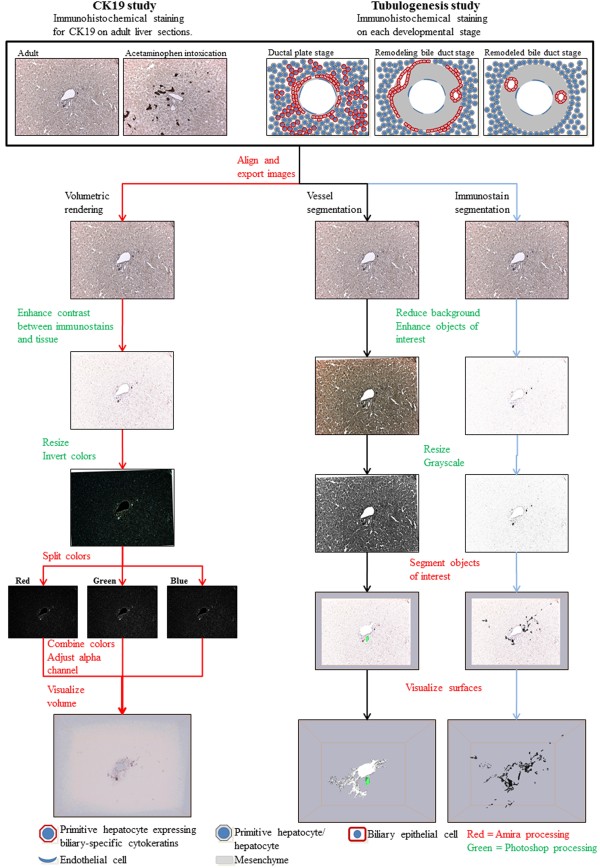
**Overview of the 3D reconstruction process**. Serial formalin-fixed and paraffin-embedded liver sections were immunohistochemically stained and photographed. The images were aligned with Amira, exported and copied to three different stacks; one for the volumetric rendering and two for segmentation of the vessels and immunostains, respectively. With the purposes of enhancing the objects of interest the images were modified with Adobe Photoshop and resized. In Amira the color channels of the image stacks for the volumetric rendering were separated followed by their recombination and calculation of a new transparency channel. Visualization was completed through the use of the Voltex module. For the segmentation of the portal vessels and immunostains, each object of interest was labeled with Amiras built-in segmentation editor followed by their visualization with the SurfaceGen and SurfaceView modules.

To reconstruct protein expression in the biliary tree two protocols were developed. The volume rendering protocol was adapted and modified from a recent protocol by Handschuh et al. [22], and the segmentation based surface rendering protocol was developed *in situ*. Essentials of the 3D-reconstruction process are depicted in Figure 1. Specimens were from formalin-fixed, paraffin-embedded human liver tissue. Fifty consecutive sections were cut from normal adult livers, from acetaminophen intoxicated adult livers, and from prenatal human livers (age 7 weeks + 2 days post conception and 15½ weeks post conception, respectively). For reconstruction of the biliary tree in normal liver and acetaminophen intoxicated liver with ductular reactions each section was stained for CK19. Of the 50 sections cut from each liver, 47 were included in the reconstruction of the normal liver, while 40 sections were included in the acetaminophen intoxicated liver. For the tubulogenesis study, every 10^th ^section was immunostained for CK7, CK19, EpCAM, SOX9, AQP1, laminin, or nestin, respectively. Each protein was therefore included 5 times in each 3D-reconstruction, representing a total of 250 μm liver in the z-direction per specimen. Of the 50 consecutive sections, 42 were used in each 3D-reconstruction.

The immunostained sections were manually digitalized in TIFF format (Tagged Image File Format) using a Leica DC300 FX CCD camera (24-bit RGB color depths, 1392 × 1040 pixels) attached to a Leica DM4000 B microscope controlled by the Leica Application Suite version 2.8.1 software (Leica, Switzerland) at 10x magnification (894 × 668 μm). The computer used for the three-dimensional image processing was equipped with an Intel^® ^XEON^® ^E5530 CPU, 48 GB DDR3 RAM, and an NVIDIA^® ^Quadro^® ^6000 graphics card. The operating system was 64 bit Microsoft Windows 7 professional (http://www.microsoft.com).

#### Image alignment

The acquired images were loaded as a stack into the Amira 5.3.3 (http://www.amira.com) image processing software using the "ColorField" import function, landmarked and manually aligned using the "AlignSlices" module and exported as 2D TIFF images.

#### Volume rendering

##### Image editing

In Adobe Photoshop CS4 (http://www.adobe.com) the aligned images were contrast and brightness enhanced to improve the contrast of the immunostainings. The background color was adjusted to pure white to ensure a homogenous background throughout the image stack. The images were color inverted, resized to 696x520 pixels/50 pixels per inch and saved in the 24-bit uncompressed TIFF format. This reduction in size led to a negligible loss in quality, but facilitated the computation of the 3D-structures in Amira.

##### Volumetric rendering

The images were reloaded as a stack into Amira, this time using the "AllChannels" import function to separate the color and transparency channels. An arbitrary voxel size of 1 × 1 × 10 was chosen in order to add visual depth to the stack, rather than creating a relatively flat 3D-structure. The red, green and blue color channels were combined using the "ColorCombine" tool, thereby automatically calculating a new alpha (transparency) channel. Standard image formats such as JPEG and TIFF are stored so each image pixel has a value in the range [0,255] and can thereby be represented by 8 bits (1 byte). The values "0", "128" and "255" represent black, grey and white, respectively. Standard color images consist of three 8-bit integers, one for each of the color channels red, green and blue, giving a total of 24 bits for describing the color of each pixel. In order to reinvert the colors of our inverted images we attached Amiras' Arithmetic tool to the resulting image stack. For reinverting the colors we therefore multiplied the color values by -1 and added the value "255" for each color channel by entering the following equations for each channel: Red channel: Ar*-1+255; green channel: Ag*-1+255; blue channel: Ab*-1+255; alpha channel: Aa. A "Voltex" module was attached to the resulting image stack for visualizing the volume. Adjustment of the alpha value made it possible to tune the transparency of the resulting volume.

#### Segmentation based surface rendering

##### Image editing

With the purpose of removing obvious artifacts, such as particles, reducing unwanted background noise, and enhancing the contrast of the hepatic vessels or the immunostainings relative to the tissue, the images were modified in Adobe Photoshop. All images were digitally copied into two series. By adjusting the contrast and brightness, tissue on one image series was darkened, so the hepatic vessels brightly stood out thereby facilitating their identification in later segmentation steps. Optionally, vascular lumina were delineated and whitened using the "Magic Wand Tool", "Paint Bucket Tool" and "Brush Tool" to reduce background in the vessels even further.

The other image series was similarly modified to enhance the contrast of the immunostainings relative to the tissue thereby facilitating their later segmentation. The identification and removal of background noise was conducted using the "Color Range" tool to identify dark unwanted background, the "Refine Edge" option to clearly delineate the background, and the "Brush Tool" and "Pencil Tool" to remove it. Following contrast and brightness enhancement and background removal, the same tools were optionally employed to further darken the immunohistochemical stains. Relatively weak CK19 and EpCAM staining were not darkened. The images were resized to 696x520 pixels/50 pixels per inch. All images were converted to grayscale and saved in JPEG format.

##### Surface rendering

The modified images were stacked in Amira 5.3.3 (http://www.amira.com) and given the voxel size 1 × 1 × 10. In "Segmentation Editor" the vessel lumina enhanced stack and the immunostained proteins of the protein enhanced stack were labeled both manually and by thresholding. Each structure (lumen or protein) was given a specific color and named. Finally, the labeled stacks were visualized in 3D using Amira's "SurfaceGen" and "SurfaceView" modules without smoothing.

## Results

### Methodological considerations

Since objects or structures that appear to be separate entities on a standard histological section may in fact be connected when observed in three dimensions several techniques for the reconstruction of 3D structure from serial 2D histological sections have been presented [22,27-29]. Here we present a novel digitized approach for high resolution 3D reconstructions of protein expression and vessel branching based on standard 2D sections [30-32]. The protocols developed are based on archival serial sectioned tissue samples applying state-of-the art image processing using Adobe Photoshop combined with the highly flexible software platform "Amira".

We employed and combined two different methods for creating 3D-reconstructions. One method involved visualizing the portal area vessels and expression of marker proteins by creating surface renderings from image segmentation, a widely used method based on the genesis of polygons of the objects of interest. However, this method is labor intensive, depends upon a tedious manual segmentation of the objects of interest and is subject to user-interpretation of what should be segmented. We managed to combine this segmentation based approach with at volume rendering protocol adapted from Handschuh et al [22]. Volume rendering is a fast technique for visualizing tissue, even in their original colors, while reducing selection bias due to subjective interpretation of the specimen by the investigator. The use of inverted colors split into separate color channels has the additional advantage that input data can be treated as grayscale images and filters for correcting section stretching can be applied. However, the volumetric rendering cannot create true three-dimensional objects, as it does not involve the formation of vectors.

### Three-dimensional reconstruction of CK19 positive structures in adult normal or acetaminophen intoxicated human liver

**Figure 2:**
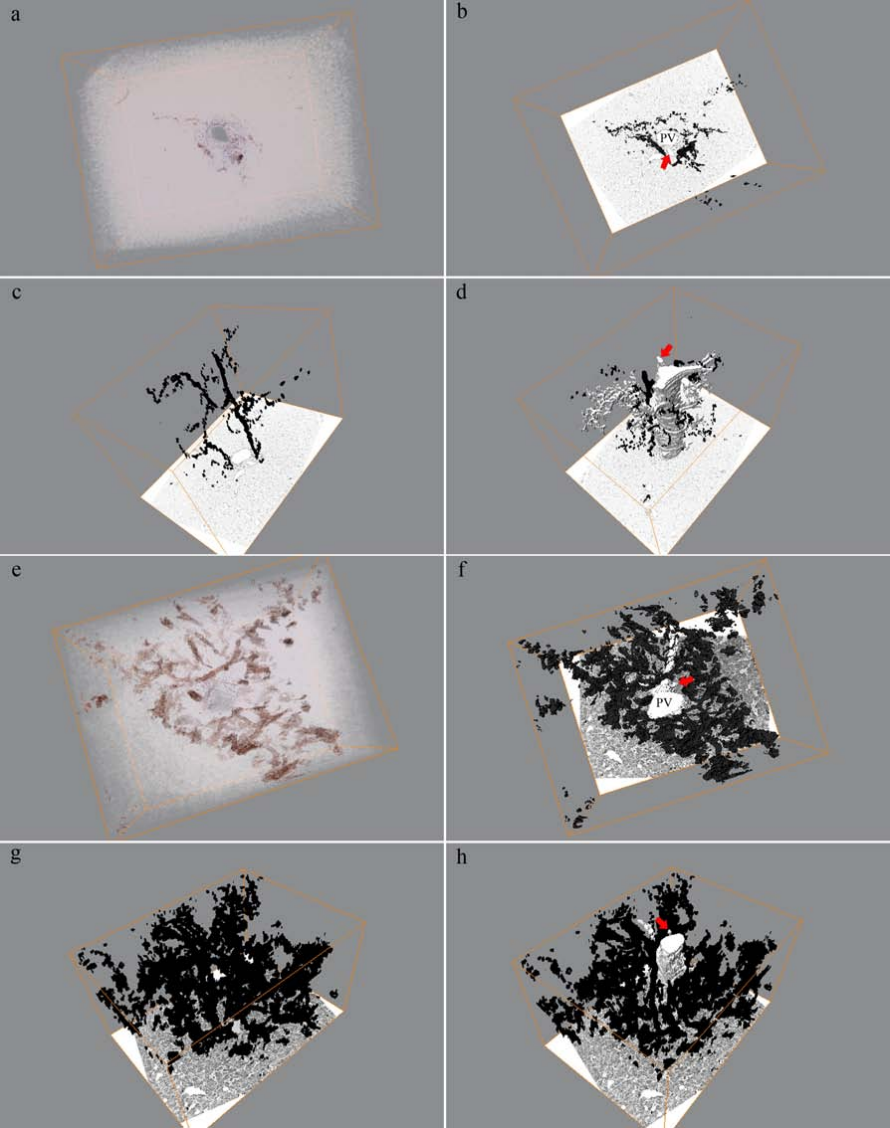
**Volumetric and segmentation based renderings of adult normal and acetaminophen intoxicated livers**. (a) Volumetric rendering of normal adult human liver optimized for CK19 presentation. (b-d) Segmentation based renderings depicting the progression of the portal vessels and the distribution of CK19. (e) Volumetric rendering of acetaminophen intoxicated liver with clear presentation of the CK19 positive ductular reaction. (f-h) Segmentation of CK19 from the same volume as (e) illustrating the complex regenerative network. (a, e) CK19 is immunostained in brown color. (b-d and f-h) CK19 is presented in black and portal vessels in white colors. Arrows point to the hepatic artery. PV = Portal vein.

The protocols for the digitized 3D-reconstruction applied the CK19 protein as unique marker of human cholangiocytes as previously done by Theise et al. [14]. Figure 2 presents the volumetric and segmentation based reconstructions of a portal area from a normal (Figure 2a-d) and an acetaminophen intoxicated (Figure 2 e-h) liver specimen, respectively. Figure 2a depicts the complete image stack visualizing the presence of cords of CK19 positive cells along the portal vein and in the parenchymal lobule. In Figure 2b-d, the segmentation based reconstruction emphasizes the distribution of the portal vein and connecting lobular vessels, the hepatic artery and the CK19 positive cells. Within this image stack the portal vein and hepatic artery present as tubular and mostly unbranched structures. However, also complex branching paths of CK19 positive cells along the portal vein and into the lobule were observed (Figure 2b-d). Few aggregations of CK19 positive cells without connection to the portal bile ducts within this image stack were additionally observed (Additional file 1).

In the acetaminophen intoxicated liver, CK19 stained not only cells in intrahepatic bile ducts but also the fine tortuous network of ductular reactions branching from the bile ducts into the hepatic parenchyma (Figure 2e-h; Additional file 1).

### Three-dimensional reconstruction of cholangiolar tubulogenesis

**Figure 3:**
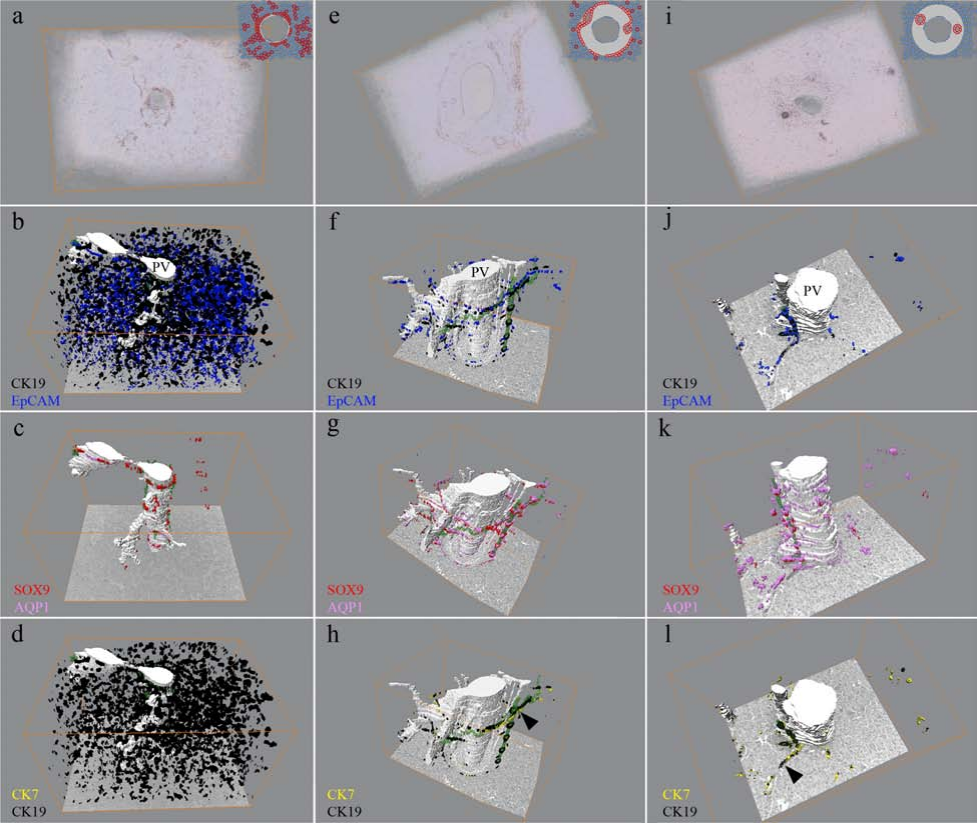
**Volumetric and segmentation based renderings of portal areas from developing liver**. Reconstructions of the "ductal plate" (a-d), "remodeling bile duct" (e-h), and "remodeled bile duct" (i-l) stages, respectively. The segmentation based renderings of the "ductal plate" stage throughout the image stack emphasize presentation of CK19 and EpCAM by the primitive hepatocytes and the ductal plates (b) whereas strong nuclear expression of SOX9 localize to the latter (c). Likewise, the branching portal veins are visible. (f-h) The investigated proteins mark the ductal plate and the remodeling bile duct, where the latter is presented as a sleeve of biliary cells surrounding the portal area. The bile ducts share lumen with the ductal plate in one end of the stack (h, arrowhead). In the adult liver (i-l) the biliary cells express the proteins of the ductal plate and make projections into the parenchyma (l, arrowhead). The biliary lumina are presented in green and the portal vessels in white colors. PV = Portal Vein.

**Figure 4:**
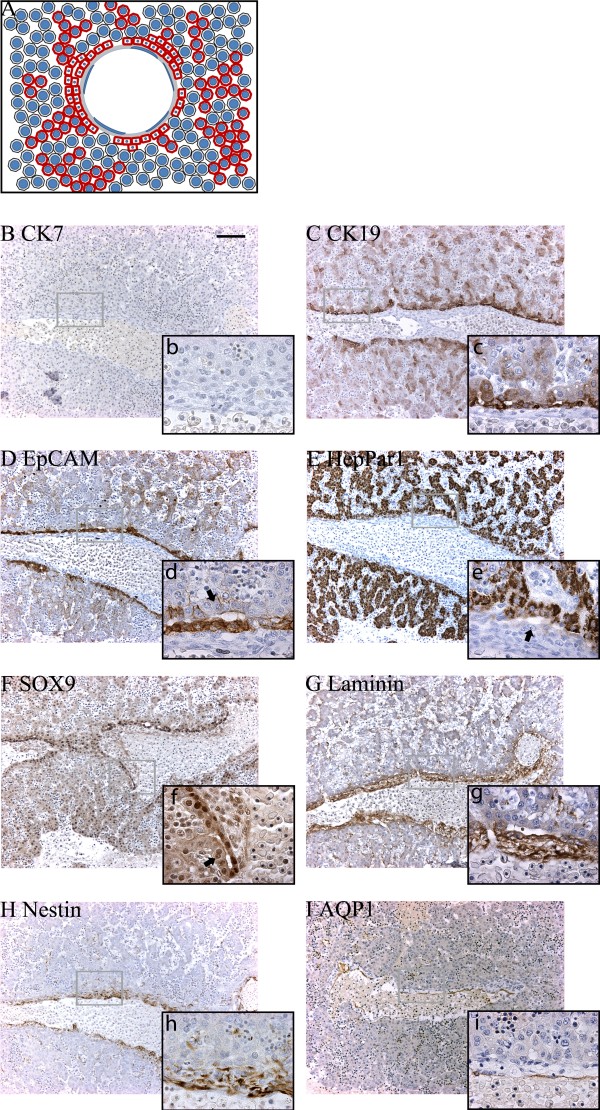
**Protein expression in the "ductal plate" stage at 5 weeks + 6 days post conception**. (A) The graphical interpretation of the "ductal plate" stage illustrates how the partly bi-layered ductal plate is surrounded by primitive hepatocytes. (B) CK7 could not be detected at this time of development. (C) CK19, (D) EpCAM, and (E) HepPar1 were expressed by both primitive hepatocytes and ductal plate cells, whereas strongest SOX9 (F) expression localized to the latter. Parts of the parenchymal (outer) ductal plate layer displayed little or no immunoreactivity for EpCAM (d - arrow) or SOX9 (f, arrow), while parts of the mesenchymal (inner) ductal plate layer displayed little immunoreactivity for HepPar1 (e, arrow). Laminin (G) and nestin (H) were predominantly present throughout the portal mesenchyma and along the sinusoids. AQP1 (I) stained the venous endothelia. Upper case letters x10, lower case letters x63. Scale bar: 100 μm (for all photos marked with upper cases).

**Figure 5:**
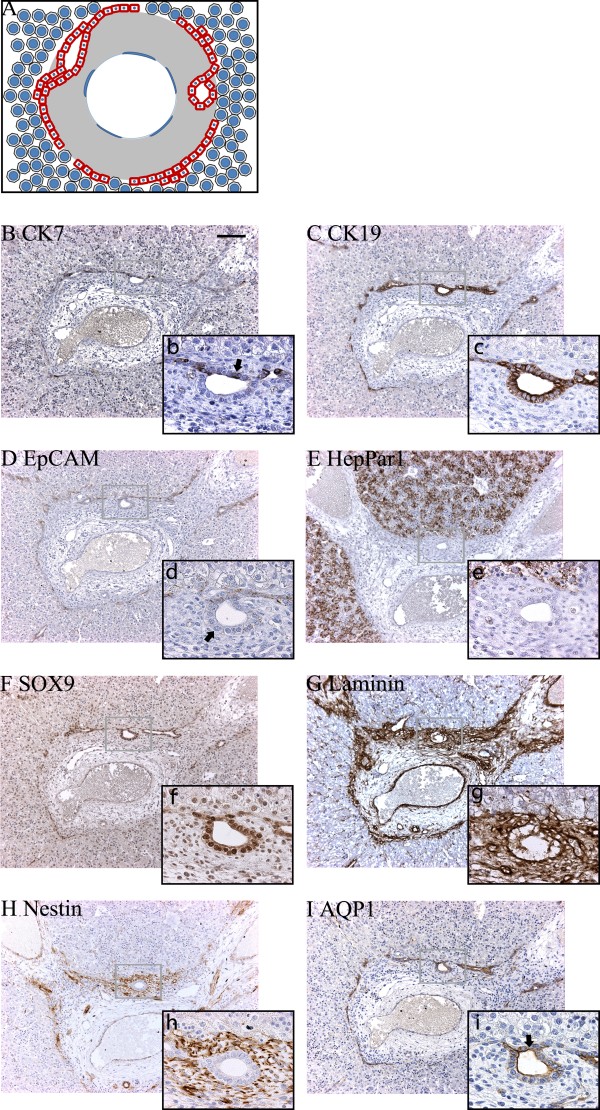
**Protein expression in the "remodeling bile duct" stage at 15½ weeks post conception**. (A). Graphical interpretation of the "remodeling bile duct" stage visualizing the formation of bile ducts from the ductal plate and their remodeling into the portal mesenchyma. (B-E) At this time in development CK7 (b, arrow) was expressed in the ductal plates and bile ducts, while the hepatocytes expressed HepPar1 (E) and were negative for CK19 (C) and EpCAM (D). Some bile ducts were only weakly positive for EpCAM (d, arrow). Expression of SOX9 (F), laminin (G) and nestin (H) was unaltered from 9½ weeks post conception. AQP1 (I) stained the biliary structures (arrows). Upper case letters x10, lower case letters x63. Scale bar: 100 μm (for all photos marked with upper cases).

**Figure 6:**
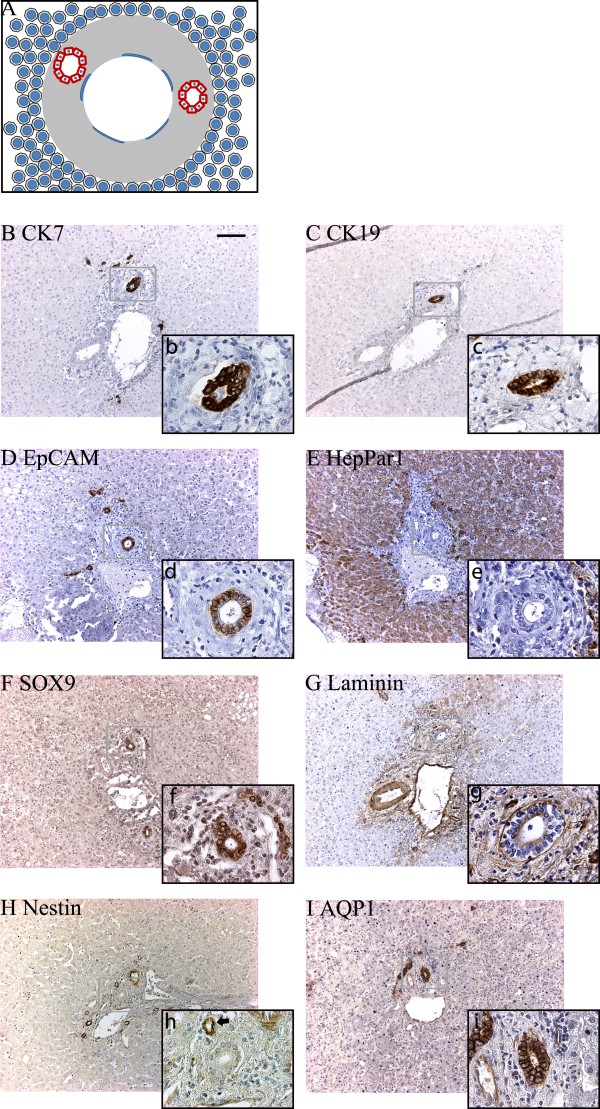
**Protein expression in adult human liver/the "remodeled bile duct" stage**. (A) The graphical interpretation illustrates that the bile ducts has fully remodeled into the portal mesenchyma, whereas the ductal plate has disappeared. CK7 (B) was now fully expressed by bile duct cells, along with CK19 (C), EpCAM (D), SOX9 (F) and AQP1 (I), whereas the hepatocytes exclusively stained for HepPar1 (E). (G) Laminin was particularly deposited around portal vascular structures, but absent from the sinusoids. (H) Nestin expression could only be found in a few portal vessels (h, arrow). Upper case letters x10, lower case letters x63. Scale bar: 100 μm (for all photos marked with upper cases).

Having established the digitized 3D-reconstruction protocols we next wanted to study the formation of the biliary tree during embryonic and fetal development of the human liver. For this purpose, the volumetric and segmentation based reconstructions of human cholangiocyte tubulogenesis using various lineage marker proteins for cholangiocyte and hepatocytes were created. However, to perform the 3D-reconstruction presented in Figure 3 the immunohistochemical staining patterns of specific hepatic lineage markers in relation to developmental stage of the intrahepatic bile ducts as well as the ages of the embryos and fetuses was characterized. A summary of the examined livers is given in Table 1. However, for simplicity the data presentation is focused to the three major developmental stages according to existing nomenclature [8-10], i.e. the "ductal plate" stage (the most primitive biliary stage) (Figure 4), the "remodeling bile duct" stage (Figure 5), and the "remodeled bile duct" stage (Figure 6).

#### The "ductal plate" stage

A schematic illustration of the "ductal plate" stage is provided in Figure 4A. The ductal plates were identifiable as a layer of cells surrounding the thin venous portal mesenchyme, separating it from the primitive hepatocytes. This layer was nearly continuous, but occasionally interrupted by vascular connections between the portal veins and the parenchyma. The ductal plates had partly duplicated into two layers facing the mesenchyme and parenchyma, respectively, and slit-like lumina had formed within these layers. The "ductal plate" stage is illustrated by detailed immunohistochemical studies on an embryonic liver specimen at 5 weeks and 6 days post conception (Figure 4) as well as in the embryonic liver at 7 weeks and 2 days post conception used for the 3D-reconstruction (Figure 3a-d). At this stage, primitive hepatocytes and ductal plates were CK19 and EpCAM positive (Figure 4C, D). The primitive hepatocytes displayed strong granular cytoplasmic HepPar1 staining (Figure 4E). Interestingly, parts of the mesenchymal ductal plate layer were HepPar1 negative while the parenchymal layer was positive (Figure 4E). The ductal plates displayed stronger expression of SOX9 relative to the primitive hepatocytes (Figure 4F). Intrahepatic bile duct development was closely coordinated with deposited laminin and nestin (Figure 4G, H). Depositions of laminin were observed throughout the portal mesenchyme and additionally surrounded cells of mainly the mesenchymal ductal plate layer and were present in some venous endothelial cells. This staining pattern was as also noticed for nestin (Figure 4G, H). In addition, laminin and nestin were present along the developing sinusoids, and weak cytoplasmic laminin reactivity was observed in the primitive hepatocytes.

Three dimensional reconstruction of the "ductal plate" stage was performed on a portal area chosen from an embryonic liver aged 7 weeks + 2 days post conception (Figure 3a-d; Additional file 2). The reconstruction revealed that in this specific portal area three distinct portal veins had a common origin at one end of the stack, while appearing as entirely separate units at the other end. In a similar fashion, the ductal plates which had a common origin around the portal vein in one end of the image stack branched out as separate entities along each diverging portal vein. Several lumina in the ductal plates were traceable. However, the reconstruction revealed that none of these lumina were connected throughout the entire stack of images (Figure 3c). While CK19 and EpCAM (Figure 3b) mark both the primitive hepatocytes and ductal plate cells through all sections in the stack, the relatively strong expression of nuclear SOX9 clearly marked only the ductal plates (Figure 3c).

#### The "remodeling bile duct" stage

A schematic illustration of the "remodeling bile duct" stage is provided in Figure 5A. This stage in development was observed in specimens from 9½ weeks post conception and throughout gestation. Large circular lumina were present between the two ductal plate cell layers forming true glandular structures at 9½ weeks. Apparently, a number of these biliary duct-like structures were in the process of remodeling into the abundant portal mesenchyme, along with their ductal plate attachments. With advancing age the remodeling bile ducts lost their attachment to the ductal plates, which progressively vanished leaving the limiting plate as the parenchymal-mesenchymal interface observed in the fully developed liver. Concurrently, arteries and vascular structures appeared in the portal mesenchyme.

A fetal liver of 15½ weeks post conception was used for detailed immunohistochemical characterization and 3D-reconstruction. At this stage, CK19, EpCAM and SOX9 were mainly expressed in ductal plates and remodeling intrahepatic bile ducts whereas the primitive hepatocytes were largely negative (Figure 5C, D, F). Interestingly, the primitive hepatocytes remained HepPar1 positive (Figure 5E). Concurrently, CK7 was detected in few cells of the migratory bile ducts and of the ductal plates (Figure 5B). Bile duct and ductal plate cells displayed AQP1 in the lateral and apical domains (Figure 5I). In the portal area, deposited laminin and nestin mainly localized to more cell dense areas of the mesenchyme while the latter additionally localized to the apical and baso-lateral domains of the epithelial cells of bile ducts and to the arterial endothelial cells (Figure 5G,H).

The 3D-reconstruction of the "remodeling bile duct stage" was performed in the portal area of a fetal liver 15½ weeks post conception (Figure 3e-h; Additional file 3). The study revealed that, as in the "ductal plate" stage, several developing portal veins shared lumen at one end of the image stack and spread out as discrete entities (Figure 3, Additional file 3). As opposed to the "ductal plate" stage, the numerous slit-like lumina in the ductal plate had largely vanished. However, through all sections, one large tubular bile duct could be traced. The 3D-reconstruction unveiled that while this bile duct was remodelled into the portal mesenchyme in one end of the image stack, it shared lumen with the ductal plate in the opposite end (Figure 3f-h). Moreover it was illustrated that through the entire image stack only the biliary cells stained positive for CK7 and CK19 (Figure 3h), EpCAM (Figure 3f) and SOX9 (Figure 3g), for which the primitive hepatocytes were negative at all levels. In addition to the portal venous endothelium, also the biliary cells stained for AQP1 throughout the stack (Figure 3g).

#### The "remodeled bile duct" stage

The "remodeled bile duct" stage, here examined in adult liver, morphologically resembled that of the "remodeling bile duct" stage with the exception that ductal plates surrounding the portal area were not observed (Figure 6). In addition, numerous biliary structures were present in the portal area and in the hepatic parenchyma, staining positive for CK7, CK19, EpCAM, SOX9 and AQP1 (Figure 6 B-D, F, I). Only hepatocytes stained for HepPar1 (Figure 6E). Laminin deposition was similar to that observed during the "remodeling bile duct" stage (Figure 6G), while nestin was found in few portal area vascular structures (Figure 6H).

The 3D-reconstruction of the remodeled stage, here illustrated with a portal area from an adult specimen, revealed the presence of a tubular portal vein that had no luminal connections to other portal areas within the image stack (Figure 3i-l, Additional file 4). The lumen of a single slender, cylindrical bile duct was traceable through all sections. At one end, the reconstruction showed the bile duct to bifurcate and send a cholangiocytic projection into the hepatic parenchyma (Figure 3j-l). Numerous other cholangiocytic projections were also observed extending from the portal area into the parenchyma; however, these carried no traceable lumen. Throughout the reconstruction, only the cholangiocytes stained for CK7, CK19, EpCAM and SOX9 (Figure 3j-l), while remnants of the ductal plate could not be identified.

## Discussion

The advantages of 3D-reconstructions became apparent when tracing CK19 positive cells in the liver. Immunohistochemical studies of CK19 expression in liver tissue often stain cells in the hepatic parenchyma, far from the portal area, which lead to the speculation that these cells represent remnants of the ductal plate, or alternatively the Canals of Hering. Manual tracing, coloring in and tabulation of CK19 positive cells in serial sectioned liver revealed that many of these cells were in fact connected and extended from the interlobular bile ducts into the hepatic parenchyma [14]. By combining our protocols for segmentation based surface rendering with volumetric rendering, we successfully extended these studies in adult human liver and visualized portal areas from a normal and an acetaminophen intoxicated liver in actual three dimensions. The volumetric renderings directly depicted that in normal liver the Canals of Hering originate from branches of CK19 positive cells connected to the portal bile ducts. The segmentation based rendering highlighted the CK19 positive cells and directly demonstrated connections between the cells in the lobule and cells of the bile ducts. The study showed that these were not isolated ductal plate remnants. However, sporadic CK19 positive cells were observed in the lobule that had no apparent connection to the portal area of interest within the investigated image stack. Likewise, the volumetric and segmentation based renderings successfully depicted the intricate network of CK19 positive ductular reactions emanating from the biliary epithelium in the portal area of acetaminophen intoxicated livers.

With the purpose of gaining an increased understanding of the mechanisms behind development of the human biliary tree we had to develop protocols for 3D-reconstructions using multiple marker proteins. Therefore, we first conducted classical immunohistochemistry on serially sectioned FFPE-liver tissue and next employed the 3D-protocols for digitally reconstructing vessels and protein expression in the portal areas throughout fetal development. Basing the investigation on previously defined markers for the biliary/progenitor (CK7, CK19, EpCAM, SOX9) and hepatocytic (HepPar1) lineages [4,23-26], we investigated human cholangiocyte tubulogenesis from 5 weeks + 6 days post conception and into adulthood. Our findings showed expression of both CK19 and HepPar1 in the ductal plates and in primitive hepatocytes during the embryonic period, corroborating previous investigations showing CK19 and HepPar1 to be early indicators of prospective liver development from the ventral endoderm [10,33,34]. Staining for CK19 on each consecutive section would have presented the ductal plate as a continuous sleeve of CK19 positive cells encircling the portal area. However, due to limited availability of prenatal human liver, only every 10^th ^section was stained for the investigated markers. Correspondingly, our study and previously published reports observed EpCAM expression in both embryonic primitive hepatocytes and ductal plate cells [24]. Whereas ductal plate cells and the primitive hepatocytes displayed triple presentation of CK19, EpCAM and HepPar1, suggesting a common origin, only cells committed to the cholangiocytic lineage strongly expressed SOX9 in their nuclei. Interestingly, we observed an asymmetric expression of these proteins in bi-layered ductal plates. Whereas some cells of the inner mesenchymal ductal plate layer were HepPar1 negative and SOX9 positive, a number of cells in the outer parenchymal ductal plate layer were only slightly positive for EpCAM in the lateral membranes. These cells did not express SOX9, a transcription factor known to be involved in chondrogenesis and male gonad development [35-37]. In line with our study of human liver development, studies of the developing mouse liver have recently shown Sox9 to be expressed initially in the mesenchymal ductal plate layer and only later in the parenchymal ductal plate layer [26]. Even though Sox9 proved to be the earliest indicator of murine cholangiocyte commitment yet identified, mice genetically deficient in Sox9 were still able to generate morphologically normal hepatic bile ducts, albeit with a delayed development. The authors concluded that in mouse livers, a new mode of asymmetric cholangiocyte tubulogenesis takes place, in which cholangiocytes associated with the mesenchyme in the ductal plate instruct neighboring primitive hepatocytes to develop into cholangiocytes forming the parenchymal ductal plate layer. Our immunohistochemical data, and a concurrent study by Raynaud et al., suggest that this remarkable asymmetrical mode of tubulogenesis also takes place in developing human liver [38].

Adult stem cells are proposed to reside in protective microenvironments, known as niches, which regulate progenitor cell fate. In the liver, the ductal plates have been hypothesized to constitute the prenatal and neonatal hepatic progenitor niche and to be antecedent to the Canals of Hering, the proposed adult progenitor niche [11,13,14,39]. However, these studies were based on human fetuses no younger than 16 weeks of gestation, corresponding to 14 weeks post conception [11,13,39]. An interesting feature of immature hepatic progenitor cells is the absence of CK7 expression. This cytokeratin is expressed as hepatic progenitor cells acquire a mature biliary phenotype, which is in accordance with the demonstration of CK7 in the later stages of hepatic organogenesis [4,40]. In our studies, the final commitment towards the biliary lineage in human liver was not observed until the fetal period at 13½ weeks post conception, at which time CK7 appeared. CK7 was expressed by a few remodeling bile duct cells, and by 14 weeks post conception sporadic CK7 positive cells additionally located to the ductal plates. With the phenotypic switch to CK7 expression, the developing biliary cells immunostained for all the proteins that were also found in adult cholangiocytes, marking their final commitment to the biliary lineage.

The digitized 3D-reconstruction of cholangiocyte tubulogenesis neatly emphasized and extended the 2D-descriptions of the protein expression patterns. The phenotypic switch displayed by the primitive hepatocytes from being CK19 and EpCAM positive during embryonic life to being negative in the fetal period was particularly evident, and so were the projections of biliary structures positive for CK7, CK19, EpCAM, SOX9 and AQP1 from the bile duct and into the hepatic parenchyma in the remodeled stage. Importantly, the 3D-reconstructions revealed details that would not have been apparent from analyzing 2D-sections alone. In both the "ductal plate" and "remodeling bile duct" stages, it became evident that portal areas that would appear to be isolated units on most sections in fact shared lumen in one end of the image stacks. Intrahepatic bile duct development is initiated at the liver hilum and gradually extends towards the periphery, resulting in more developed bile ducts at the hilum and less developed structures in the periphery [41]. This process was in effect visualized in the 3D-reconstruction of the remodeling bile duct stage. The 3D-reconstruction clarified that while the remodeling bile duct shared lumen with the ductal plate in one end of the image stack, the duct remodeled into the portal mesenchyme in the other end. Therefore this stack actually represented several stages in the development of the intrahepatic biliary tree.

## Conclusions

In conclusion, our two different approaches to digitizing 3D-reconstructions allowed the visualization of hepatic protein expression patterns and the spatial relationship between hepatic vessels during human embryonic and fetal liver development and in human liver disease exemplified by acetaminophen intoxication. The presented protocols can easily be applied to future studies in directly unraveling the complex tissue architecture of liver and other organs in health and disease.

## Authors' contributions

PSV, KM, and HCB conceived the idea of the study. KM and HCB obtained the embryonic and fetal human livers. BV and BQ obtained the adult human livers. PSV, PJ and BMH optimized and performed the immunohistochemistry. PSV developed and performed the computer-assisted three-dimensional reconstructions. All authors contributed to the writing of the paper and approved the final version.

## Supplementary Material

Additional file 1 **3D rendering of adult normal and acetaminophen intoxicated livers**. This video shows the rotating volumetric (left panels) and segmentation based (right panels) renderings of CK19 in the portal areas from a normal liver (top panels) and an acetaminophen intoxicated liver (lower panels). Colors as for Figure 2 Click here for file

Additional file 2 **3D rendering of a hepatic portal area in the "ductal plate" stage**. This video shows the 3D renderings based on serial sections of an immunohistochemically stained hepatic portal area from a human embryo at 7 weeks and 2 days following fertilization. Colors as for Figure 3. Click here for file

Additional file 3 **3D rendering of a hepatic portal area in the "remodeling bile duct" stage**. This video shows the 3D renderings based on serial sections of an immunohistochemically stained hepatic portal area from a human fetus at 15½ weeks following fertilization. Colors as for Figure 3. Click here for file

Additional file 4 **3D rendering of a hepatic portal area in the "remodeled bile duct" stage**. This video shows a 3D rendering based on serial sections of an immunohistochemically stained hepatic portal area from an adult human. Colors as for Figure 3. Click here for file
